# Multiple phytohormones promote root hair elongation by regulating a similar set of genes in the root epidermis in Arabidopsis

**DOI:** 10.1093/jxb/erw400

**Published:** 2016-10-31

**Authors:** Shan Zhang, Linli Huang, An Yan, Yihua Liu, Bohan Liu, Chunyan Yu, Aidong Zhang, John Schiefelbein, Yinbo Gan

**Affiliations:** ^1^Zhejiang Key Laboratory of Crop Germplasm, Department of Agronomy, College of Agriculture and Biotechnology, Zhejiang University, Hangzhou, China; ^2^Department of Molecular, Cellular, and Developmental Biology, University of Michigan, Ann Arbor, MI, USA

**Keywords:** *Arabidopsis thaliana*, auxin, cytokinin, ethylene, phytohormones, root hair, tip growth.

## Abstract

Multiple phytohormones, including auxin, ethylene, and cytokinin, play vital roles in regulating cell development in the root epidermis. However, their interactions in specific root hair cell developmental stages are largely unexplored. To bridge this gap, we employed genetic and pharmacological approaches as well as transcriptional analysis in order to dissect their distinct and overlapping roles in root hair initiation and elongation in *Arabidopsis thaliana*. Our results show that among auxin, ethylene, and cytokinin, only ethylene induces ectopic root hair cells in wild-type plants, implying a special role of ethylene in the hair initiation stage. In the subsequent elongation stage, however, auxin, ethylene, and cytokinin enhance root hair tip growth equally. Our data also suggest that the effect of cytokinin is independent from auxin and ethylene in this process. Exogenous cytokinin restores root hair elongation when the auxin and ethylene signal is defective, whereas auxin and ethylene also sustain elongation in the absence of the cytokinin signal. Notably, transcriptional analyses demonstrated that auxin, ethylene, and cytokinin regulate a similar set of root hair-specific genes. Together these analyses provide important clues regarding the mechanism of hormonal interactions and regulation in the formation of single-cell structures.

## Highlight

Auxin, ethylene, and cytokinin regulate root-hair initiation unequally but promote elongation synergistically. Detailed analyses suggest that these hormones regulate a similar set of root hair-specific genes through diverted upstream signaling pathways.

## Introduction

Plants have evolved flexible but finely controlled developmental regulatory networks, ensuring adaptation to changeable environments, variable habitats, and multiple stresses. External and internal signals are integrated by various plant hormones, which play a prominent role in plant developmental regulation (Wolters and Jurgens, 2009). The regulatory functions of a specific phytohormone, such as auxin, ethylene, cytokinin, and gibberellin, on multiple developmental processes have been well studied. Recent studies have been shifting to the more complex phenomenon of crosstalk between two and more hormones, as it has become evident that phytohormone pathways interact with one another to regulate various developmental processes in plants (Santner and Estelle, 2009). Most of the current studies focus on hormonal interactions at the tissue and organ level, such as shoot and root meristem cells (Durbak *et al.*, 2012). In contrast, the hormonal interactions involved in the formation of single-cell structures, such as root hairs and trichomes, have not yet been fully defined.

Root hair development is finely controlled by multiple phytohormones. Root epidermal development in Arabidopsis also provides a useful experimental model for the study of plant cell differentiation and phytohormone regulation, due to its technical advantages and its elegant developmental patterns (Grierson *et al.*, 2014). According to current models, root hair cell development can be divided into three relatively independent stages, namely cell fate determination, initiation, and elongation (Ishida *et al.*, 2008; Bruex *et al.*, 2012; Grierson *et al.*, 2014).

The Arabidopsis root epidermis is comprised of two types of cells, root hair cells and non-hair cells, whose spatial distribution follows a position-dependent mechanism (Duckett *et al.*, 1994). Root hair cells locate over the intercellular space between two underlying cortical cells (the ‘H’ position), whereas non-hair cells are present over a single cortical cell position (the ‘N’ position). After committing to ‘H’ cells, root hair cells undergo gene expression reprogramming and cell wall morphological changes, leading to root hair initiation (Berger *et al.*, 1998). The root hair initiation is followed by tip growth, a process featuring the rapid elongation of the root hair tube (Ishida *et al.*, 2008).

Extensive forward and reverse genetic studies have identified a large collection of genes that function in each of the developmental stages (Grierson *et al.*, 2014). Root epidermis fate determination is mainly regulated by two groups of transcription factors, namely the non-hair fate promoters and the root hair fate promoters, respectively (Grierson *et al.*, 2014). Many other cell wall- and cytoskeleton-related functional genes are involved in the initiation and elongation steps, and loss-of-function mutations in these genes results in root hair initiation defects or tip growth arrest (Baumberger *et al.*, 2001; Cho and Cosgrove, 2002).

Importantly, root hair development is also regulated by phytohormones. Auxin and ethylene are the two best-characterized positive regulators in root hair morphogenesis, as evidenced by genetic and pharmacological analyses. Several auxin- and ethylene-insensitive mutants, including *aux1*, *axr1*, *axr2*, *etr1*, and *ein2*, exhibit a short-hair phenotype (Masucci and Schiefelbein, 1996; Rahman *et al.*, 2002). Further, treatment of seedlings with the ethylene precursor aminocyclopropane carboxylic acid (ACC) and an auxin indole-3-acetic acid (IAA) promote root hair elongation significantly, whereas the ethylene biosynthesis inhibitor aminovinylglycine (AVG) inhibits root hair elongation (Pitts *et al.*, 1998). In contrast, the role(s) of auxin and ethylene in initiation are controversial. Although the auxin-response mutants *aux1* and *axr1* and the ethylene-response mutants *etr1* and *ein2* present normal root hair density, both the auxin- and ethylene-insensitive *axr2* mutant and the *aux1 etr1* double-mutant exhibit reduced root hair density (Masucci and Schiefelbein, 1996).

In addition to auxin and ethylene, our previous research revealed cytokinin as a novel root hair development regulator (An *et al.*, 2012). Application of exogenous cytokinin promotes root hair elongation while lines that overexpress a cytokinin oxidase exhibit a short-hair phenotype (An *et al.*, 2012). The signaling of ethylene and cytokinin is integrated by ZINCFINGERPROTEIN 5 (ZFP5), which is a newly characterized C2H2zinc finger protein (An *et al.*, 2012).

Taken together, there is substantial evidence suggesting that root hair development in Arabidopsis is controlled by auxin, ethylene, and cytokinin simultaneously. Unfortunately, the interactional relationship among these phytohormones in the root epidermis remains undetermined. This means that, although the root hair phenotypes of phytohormone mutants and exogenous hormone-treated plants are well documented, the molecular basis of the regulatory roles of phytohormones remains largely unclear.

To this end, we sought to resolve the roles of these phytohormones in Arabidopsis root hair initiation and elongation in this study. Our results demonstrate that only ethylene is able to induce ectopic root hair cells in wild-type plants and to partially rescue the phenotype of *cpc*, a hairless mutant. In root hair elongation, auxin, ethylene, and cytokinin enhance tip growth similarly. Transcriptome data from quantitative PCR and public microarray datasets indicates these three phytohormones activate a similar set of root hair-specific genes at the root epidermis.

## Materials and methods

### Plant growth conditions

The wild-type control used in this study was *Arabidopsis thaliana* ecotype Col-0. For most experiments, seeds were surface-sterilized for 10 min in 30% bleach solution and rinsed five times with sterile distilled water. After vernalization in the dark for 2 d at 4°C, the seeds were germinated and grown vertically on Murashige and Skoog (MS) plates in a growth chamber, as described previously (An *et al.*, 2012). Growth conditions in the chamber were controlled as follows: 22 ± 2°C, 90–120μmol m^–2^s^–1^, 16h light/8h dark, 68–78% humidity.

### Root hair density and length measurement

The root hair pattern was determined using Masucci and Schiefelbein’s method with minor modifications (Masucci and Schiefelbein, 1996). Briefly, root hairs of the primary root on 7-d-old seedlings were visualized using a Leica M295 dissecting microscope (Leica Microsystems). For each seedling root, two regions were randomly chosen in the mature root hair zone. In each region, the root hair number of five consecutive H position epidermal cells from the same cell file were counted. The root hair number of adjacent five consecutive N position epidermal cells were also counted. About 12 seedlings per treatment or genotype were scored, giving a total of 120 cells (5 cells × 2 locations × 12 plants) in both the N and H positions. Any protrusion was taken as being a root hair, regardless of the length. The percentage of root hair cells in the two different positions was calculated accordingly.

The procedure for determining root hair length was performed as described previously (An *et al.*, 2012). The root hair region approximately 4 mm from the root tip was recorded using a Nikon N995 digital camera. The length of 20 randomly chosen root hairs in each picture was measured using ImageJ (http://imagej.nih.gov/ij/). For each treatment or genotype, at least 16 seedlings were measured and the experiments were repeated at least twice, with similar results.

### Phytohormone treatment

Seeds are germinated on MS plates for 2 d and then transferred to hormone-containing plates for 5 d growth. Auxin indole-3-aceticacid-IAA (Sigma-Aldrich), ethylene biosynthesis precursor 1-aminocyclopropane-1-carboxylic acid (ACC) (Sigma-Aldrich), cytokinin 6-benzylaminopurine (BA) (Sigma-Aldrich), and the ethylene biosynthesis inhibitor aminoethoxyvinylglycine (AVG) (Sigma-Aldrich) were applied to plates as described previously (An *et al.*, 2012).

### RNA isolation and quantitative RT-PCR analysis

Total RNA was extracted from the roots of 7-d-old seedlings using a TRIzol kit (Invitrogen) and reverse-transcribed with M-MLV transcriptase. Samples of 1.5μg total RNA were applied in 25 μl of reaction mixture. Real-time PCR reactions were performed on a Bio-radCFX96 platform as described previously (An *et al*., 2012). Reaction mixtures were prepared as follows: 0.5 µl cDNA, 12.5 µl SYBR-green PCR mix (Takara),0.5 µl primer, and 11 µl ddH_2_O. ACTIN2 was used as a control, and three replicates were performed for each gene. The ∆∆Ct method was used to calculate the relative transcript abundance, as described previously (Zhou *et al.*, 2011). All the primers used are listed in Supplementary Table S1 at *JXB* online.

### Public microarray data and bioinformatics analysis

Public microarray data sets were downloaded from Gene Expression Omnibus (GEO, http://www.ncbi.nlm.nih.gov/geo/) under the accession numbersGSE30547 and GSE32087. Transcript abundance difference>2, false discovery rate (FDR)<0.05, and *P*<0.01 were used as the thresholds to judge the significance of gene expression differences. Gene ontology (GO) and enrichment analysis was performed on agriGO (http://bioinfo.cau.edu.cn/agriGO/index.php) with the TAIR10 database. The whole genome was considered as the background in the analysis.

### Statistical analyses

All data presented in the figures were analyzed for significance by means of ANOVA by using the GraphPad Prism software (http://www.graphpad.com/). Differences between means were determined using Student’s *t*-test, as described previously (Gan *et al.*, 2010; Zhou *et al.*, 2011).

## Results

### Ethylene plays a unique role in promoting root hair initiation

**Fig. 1.**
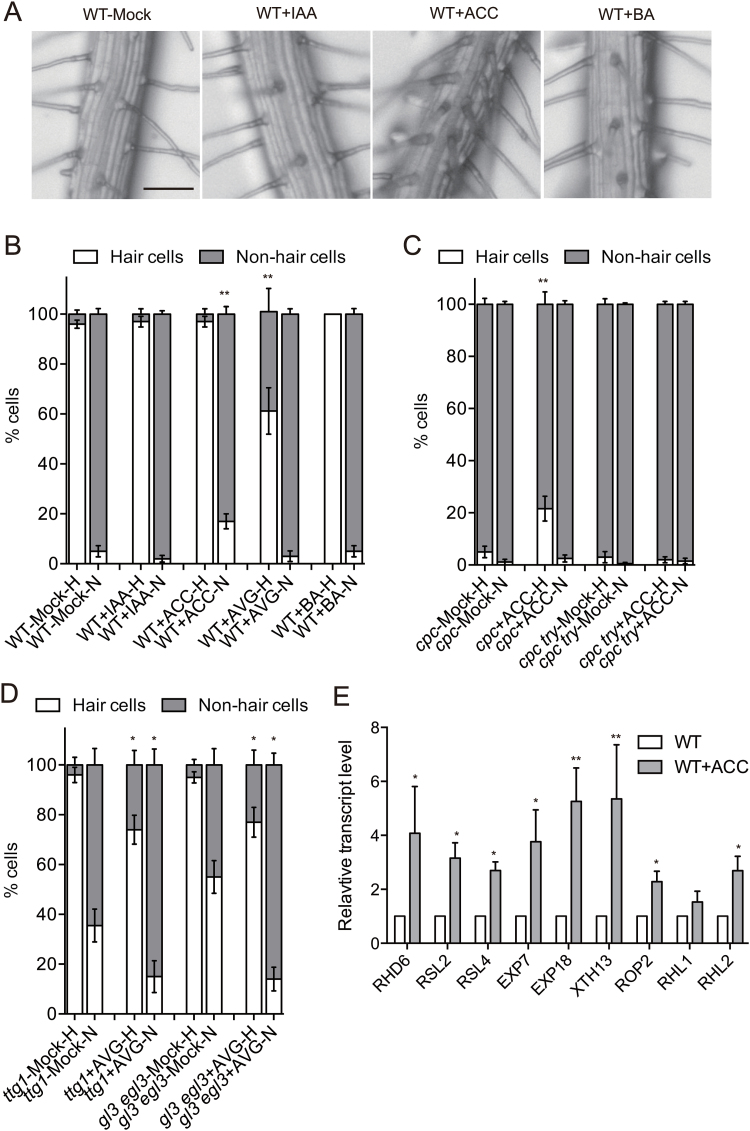
Effects of exogenous auxin, ethylene, and cytokinin on root hair density. (A) High-magnification view of the root hair region from representative wild-type seedlings under different hormonal treatments as indicated. Scale bar= 100 μm. Auxin indole-3-acetic acid (IAA, 100nM), ethylene precursor 1-aminocyclopropane-1-carboxylic acid (ACC, 1μM), cytokinin 6-benzylaminopurine (BA,100nM) were used; ‘Mock’ indicates the control. Unless stated otherwise, the same concentrations also apply to the other parts of this figure. (B)The percentage of root hair cells and non-hair cells was calculated for the ‘H’ and ‘N’ position cells. The ethylene biosynthesis inhibitor aminoethoxyvinylglycine (AVG, 100nM) was applied as one of the treatments. (C) Root hair density of mutants was analyzed under the different treatments as indicated. (D) The expression levels of characterized root hair initiation regulators were determined by qRT-PCR in wild-type seedlings with or without ACC treatment. Error bars are ± standard deviation (SD). Significant differences in (B–E) were determined using Student’s *t*-test: *, *P*<0.05; **, *P*<0.01; n.s., non-significant.

Because epistasis analysis has shown that hormones act downstream of cell fate determination (Masucci and Schiefelbein, 1996), we concentrated our study on the role of hormones in root hair initiation and elongation. We started by treating wild-type seedlings with exogenous auxin (IAA), an ethylene precursor (ACC), or cytokinin (BA) (Fig. 1A). Consistent with previous reports, auxin and cytokinin treatments failed to alter the root hair distribution pattern, whereas the ethylene precursor significantly induced ectopic root hair formation at the N positions, indicating that ethylene plays a unique role in regulating root hair initiation (Fig. 1A, B) (Masucci and Schiefelbein, 1996). This hypothesis was reinforced by the observation that H position root hairs were partially abolished by AVG, an ethylene synthesis inhibitor (Fig. 1B) (Masucci and Schiefelbein, 1996).

To further reveal the role of ethylene, we then determined its effect on root hair initiation when the root epidermal cell differentiation pattern was disrupted. *CAPRICE* (*CPC*) and *TRIPTYCHON* (*TRY*) are the principal positive regulators of root hair formation. Both *cpc* and *try* mutants produce a reduced number of root hairs (Schellmann *et al.*, 2002). In contrast, *GLABRA3* (*GL3*), *ENHANCEROFGLABRA3* (*EGL3*), and *TRANSPARENT TESTAGLABRA* (*TTG*) encode transcription factors that promote the non-hair fate of cells, and the mutants of these genes exhibit a ‘hairy’ phenotype (Galway *et al.*, 1994; Bernhardt *et al.*, 2003). Interestingly, exogenous ethylene treatment significantly induced root hair formation in the *cpc* mutant at the H positions but not in the *cpc try* mutant, indicating that ethylene induced root hair initiation only when the CPC-TRY regulatory complex was functional or at least partially functional (Fig. 1C). This also implies that inhibition of root hair initiation is more robust in the *cpc try* mutant. Blocking ethylene synthesis by AVG in the *ttg1* and *gl3 egl3* mutants inhibited root hair formation at the H and N positions equally (Fig. 1D).

To further understand how ethylene promotes root hair initiation, we determined the expression of several previously characterized root hair initiation regulators in seedlings treated with the ethylene biosynthesis precursor ACC (Fig. 1E). In agreement with the ectopic root hair phenotype, ACC treatment induced the expression of multiple key positive regulators of hair initiation, such as RHD6, RSL2, and RSL4 (Masucci and Schiefelbein, 1994; Yi *et al.*, 2010). Several other genes, including two expansins and cell wall remodeling enzymes, were also significantly up-regulated to facilitate root epidermal remodeling (Fig. 1E) (Vissenberg *et al.*, 2001; Cho and Cosgrove, 2002). Our data collectively suggest that ethylene plays a unique role in promoting root hair initiation after cell fate determination.

### Fate determination factor mutants are less sensitive to hormone treatment

**Fig. 2.**
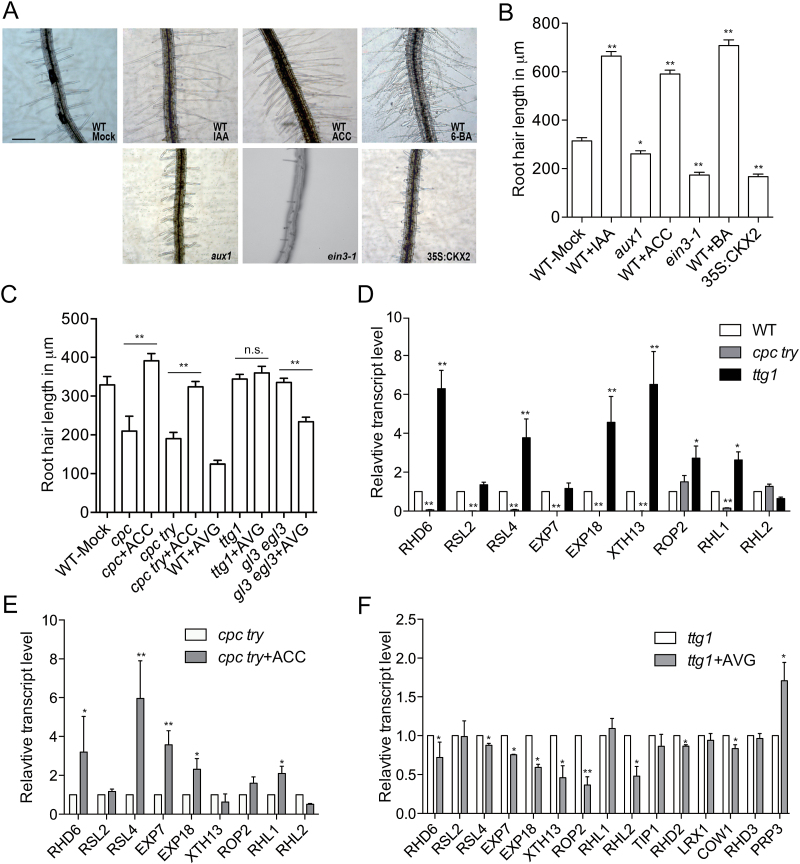
Effects of exogenous auxin, ethylene, and cytokinin on root hair elongation. (A) Representative images of the root hair region from 7-d-old seedlings under different treatments are shown. Scale bar = 200μm. ‘Mock’ indicates the control. (B, C) The average root hair lengths of different lines under the different treatments. Error bars are ±SD. (D–F) Transcript levels of characterized root hair tip growth regulators as determined by qRT-PCR in seedlings. The genetic background and treatments are indicated. Error bars are ±SD. (This figure is available in colour at *JXB* online.)

After defining the roles of auxin, ethylene, and cytokinin in initiation, we set out to determine their roles in elongation. These three phytohormones have been reported to enhance root hair elongation (Pitts *et al.*, 1998; An *et al.*, 2012). In agreement with previous reports, IAA, ACC, and BA nearly doubled the root hair length of treated seedlings in our experiment (Fig. 2A, B) (Pitts *et al.*, 1998). In contrast, abnormal short-hair phenotypes were observed in the auxin- and ethylene-response mutant *aux1*and in the ethylene downstream signaling mutant *ein3-1*and the cytokinin enzyme CKX2 over-expression line (Fig. 2A, B, Supplementary Fig. S1) (Pickett *et al.*, 1990; Chao *et al.*, 1997; Werner *et al.*, 2003).

Previous studies have been centered on the root hair density of fate determination factor mutants such as *cpc* and *cpc try*, and did not address their root hair length phenotype (Schellmann *et al.*, 2002). Here, we observed that the remaining root hairs of the *cpc* and *cpc try* mutants were significantly shorter than the hairs of the wild-type (Fig. 2C). Exogenous phytohormone treatments increase the overall length of the hairs of the *cpc* and *cpc try* mutants but with an attenuated magnitude (Fig. 2C and Supplementary Fig. S2). In addition, the ethylene biogenesis inhibitor AVG failed to shorten the root hair length of the hairy mutant *ttg1* and only slightly inhibited the root hair elongation of the *gl3 egl3* double-mutant (Fig. 2C). These observations suggest that these hairy and hairless mutants are less sensitive to perturbation of the phytohormone signals.

Next, we investigated the mechanism that underlies the decreased hormone sensitivity in these mutants. Root hair development is regulated by transcription factors and requires the coordination of cell morphogenesis, such as cell wall remodeling (Bruex *et al.*, 2012). Hence, we hypothesized that even though fate specification factors are not directly involved in elongation, they set a scenario for proper root hair elongation. To test this idea, we assayed the gene expression level of multiple regulators of tip growth in the hairless mutant *cpc try* and the hairy mutant *ttg1*.Among these genes, RHD6, RSL2, and RSL4 are the key transcription factors that regulate a group of root hair development-related genes (Masucci and Schiefelbein, 1994; Yi *et al.*, 2010). Other genes such as *EXP7* and *ROP2* are directly required for biological processes such as cell wall formation and modeling (Cho and Cosgrove, 2002; Jones *et al.*, 2002). In line with their biological functions, most of the genes exhibited extremely low transcript abundance in the *cpc try* mutant and an increased abundance in the *ttg1* mutant compared with the wild-type (Fig. 2D). After exogenous ethylene treatment, the expression level of the same gene set was significantly increased in the *cpc try* mutant (Fig. 2E). In the hairy mutant *ttg1*, root hair genes responded to the ethylene biogenesis inhibitor differently. Genes required for initiation such as *EXP7*, *EXP18*, and *XTH13* were inhibited whereas elongation-related genes such as *TIP1*, *COW1*, and *RHD3* were expressed normally, which tightly correlated with the phenotype of the *ttg1*plants under AVG treatment (Figs 1D and 2C, F) (Grierson *et al.*, 1997; Cho and Cosgrove, 2002; Böhme *et al.*, 2004). Therefore, the decrease in hormone sensitivity of the hairless and hairy mutants could be explained by the altered basal level and the changed expression responsiveness of the root hair genes upon treatment with the phytohormones.

### Cytokinin promotes root hair elongation independent from auxin and ethylene

Auxin, ethylene, and cytokinin are known to enhance root hair elongation, but we know much less about their interactions and crosstalk. It is possible that the function of one hormone requires the collaboration of other hormones, or that the action of different hormones is independent of each other.

**Fig. 3.**
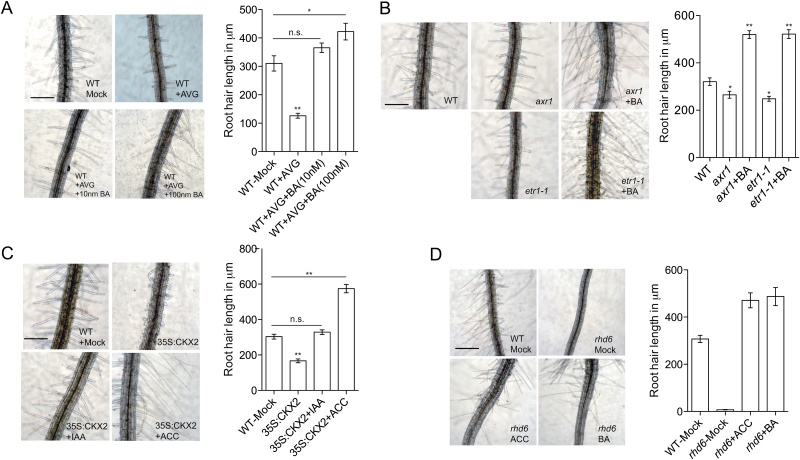
Cytokinin promotes root hair elongation independent of auxin and ethylene. (A) Wild-type seedlings were treated with the ethylene biogenesis inhibitor AVG either alone or together with low concentration (10nM) and moderate concentration (100nM) of BA. Images of representative seedlings together with average root hair lengths are shown. Error bars are ±SD. ‘Mock’ indicates the control. (B) The auxin-insensitive mutant *axr1* and the ethylene insensitive mutant *etr1-1* were treated with cytokinin BA. Images of representative seedlings together with average root hair lengths are shown. (C) The cytokinin enzyme CKX2 overexpression line was treated with IAA and ACC, and root hair length was determined. (D) The root hair defective mutant *rhd6* was treated with ACC and BA. Images of representative seedlings together with average root hair lengths are shown. Scale bars = 200μm. (This figure is available in colour at *JXB* online.)

As cytokinin has not been so well characterized previously, we centered our study on the interactions between cytokinin and the other two hormones, ethylene and auxin. It is known that cytokinin can enhance ethylene production by increasing the stability of ACC synthase (ACS), the rate-limiting enzyme in ethylene biosynthesis (Chae *et al.*, 2003; Hansen *et al.*, 2009). To evaluate the potential indirect effect of cytokinin, we treated wild-type seedlings with exogenous cytokinin BA in the presence of the ACS-specific inhibitor AVG. We observed that a relatively low concentration (10nM) of BA robustly restored the root hair length of AVG-treated plants to the level of the wild-type line (Fig. 3A). We further applied BA to the ethylene-insensitive mutant *etr1-1* and found that it nearly doubled the root hair length (Fig. 3B) (Chang *et al.*, 1993). A similar result was obtained when the auxin-insensitive mutant *axr1* was treated with BA (Fig. 3B) (Lincoln *et al.*, 1990).

On the other hand, exogenous auxin and ethylene also restored the short-hair phenotype of the cytokinin enzyme overexpression line (Fig. 3C). In addition, to confirm that the root hair defects of the 35S:CKX2 line were specifically caused by cytokinin deficiency, we supplied these plants with cytokinin oxidase-/dehydrogenase-insensitive cytokinin 6-benzylaminopurine (BA) and found that the defects could be fully rescued (Supplementary Fig. S3) (Armstrong, 1994). These results collectively suggest that cytokinin is able to promote root hair elongation in the absence of auxin and ethylene signals.

### Phytohormones enhance root hair elongation synergistically

After analyzing the biological effects of the phytohormones on elongation, we next explored their downstream targets. Both auxin and ethylene act downstream of transcription factor RHD6, because exogenous auxin and ethylene are able to restore the root hair defects of the *rhd6* mutant (Masucci and Schiefelbein, 1994). We found that exogenous cytokinin was able to restore the root hair length of the *rhd6* mutant in the same way that ethylene did (Fig. 3D). These results indicated that the three hormones might share some common target genes.

**Fig. 4.**
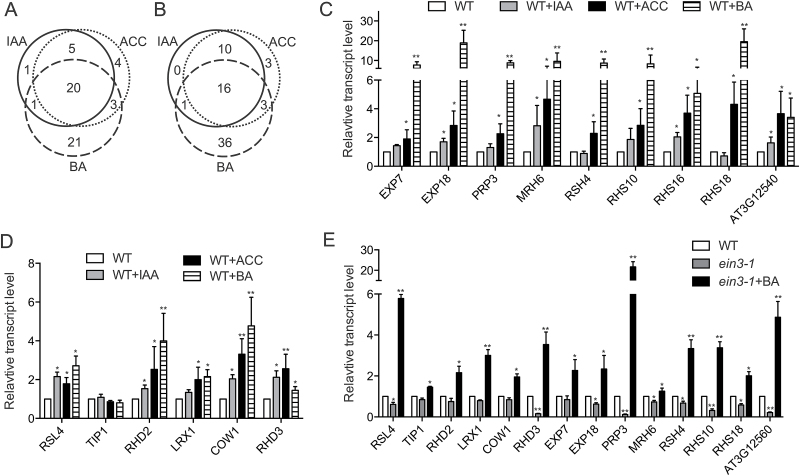
Auxin, ethylene, and cytokinin promote root hair elongation by inducing similar sets of genes. (A) Venn diagram that showing the overlapping relationship of genes that were positively regulated by auxin, ethylene, and cytokinin. The number of genes with the Root Hair Element (RHE) in their promoter region are shown. (B) Similar to (A), the number of genes with the GO annotation ‘Root hair cell differentiations’ (GO:0048765) are shown. (C) The transcript levels of genes that were regulated by all three phytohormones were determined by qRT-PCR. Only genes with the GO:0048765 annotation and that have the RHE in their promoter were selected. (D) The gene expression levels of several other predicted common targets were determined by qRT-PCR. (E) The transcript levels of phytohormone-regulated genes were determined in the *ein3-1* line with or without endogenous BA treatment.

To test this hypothesis, we compared several publically available microarray datasets to identify genes that are regulated by these hormones in the root epidermis. Genes that are positively regulated by auxin and ethylene were pooled from two microarray datasets in which RNA samples were collected from seedlings after IAA and ACC treatment (Bruex *et al.*, 2012). Because of the lack of a similar dataset for cytokinin, down-regulated genes in a cytokinin-deficient mutant were used (Nishiyama *et al.*, 2012). Two bioinformatics approaches were then applied to systematically identify genes that function in root hair development. In the first method, because a cis-regulatory element (Root Hair Element, RHE) is found in the promoters of many root hair-specific genes, we identified differentially expressed genes with this element and analyzed them for overlapping patterns (Kim *et al.*, 2006). As shown in the Venn diagram in Fig. 4A, consistent with our hypothesis, more than half of the genes were co-regulated by at least two hormones. More interestingly, more than one-third of the genes were regulated by all of the three phytohormones, indicating that this group of genes act with a ‘core function’ in root hair development (Fig. 4A). In our second analysis, genes with the GO annotation ‘Root hair cell differentiation’ (GO:0048765) were extracted and analyzed in a similar manner. Coincidently, this analysis produced a very similar overlap pattern (Fig. 4B). We also noticed that a larger number of genes were exclusively regulated by BA. This result implied that cytokinin might have a wider spectrum of target genes, but it is also plausible that unequal sensitivities of different microarray experiments may contribute to the difference.

To further confirm the interaction pattern, we determined the gene expression levels of target genes common to all three of the phytohormones by real-time PCR. Genes with the GO:0048765 annotation and that have the RHE in their promoter were selected initially. We found that the ACC and BA treatments induced the expression of most target genes that were identified by microarray, whereas IAA generated a relatively modest induction effect (Fig. 4C). We also assayed the expression levels of several other characterized root hair elongation-related genes. A similar pattern of transcription up-regulation was observed for these genes upon exogenous phytohormone treatment (Fig. 4D). It should be noted that cytokinin exerted the most profound transcriptional induction effect, although the same concentration of treatment promoted a similar extent of elongation as auxin and ethylene. To further confirm that ethylene and cytokinin could act separately, we assayed the expression level of root hair elongation-related genes in the ethylene-signaling mutant*ein3-1* upon treatment with BA. Our analysis showed that BA treatment induced the expression of most root hair-related genes in the *ein3-1* mutant, even though the expression of many genes was repressed in this line (Fig. 4E).

Root hair development is governed by multiple transcription factors. In these common target genes of hormones, *RSL4* encodes a helix-loop-helix transcription factor (Yi *et al.*, 2010), And microarray studies suggested the majority of the common target genes were positively regulated by this transcription factor RSL4 (Yi *et al.*, 2010). The same study also showed that auxin promoted root hair elongation by regulating the expression of *RSL4* (Yi *et al*., 2010). Here, we found that the expression of *RSL4* could also be induced by exogenous ethylene and cytokinin (Fig. 4D). This observation supports the possibility that RSL4 incorporates signals from multiple phytohormones and regulates root hair elongation.

**Fig. 5.**
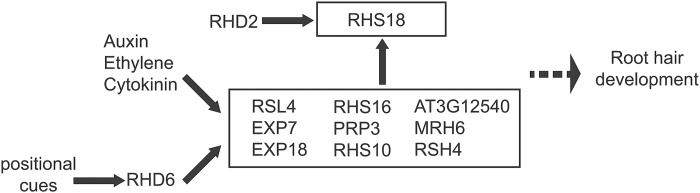
A proposed model for how phytohormones regulate root hair differentiation. This predicted regulation model is based on our qRT-PCR data and multiple microarray datasets (Yi *et al.*, 2010; Bruex *et al.*, 2012; Nishiyama *et al.*, 2012).

To predict the regulation relationship of these genes, we propose a working model based on the qRT-PCR data and several published microarray datasets (Fig. 5) (Yi *et al.*, 2010; Bruex *et al.*, 2012). According to these transcriptional data, phytohormone signals and positional cues are integrated to regulate root hair development. Taken together, our data suggest that the phytohormones auxin, ethylene, and cytokinin synergistically promote root hair elongation by regulating a group of genes that largely overlap.

## Discussion

At the root epidermis of Arabidopsis, cells differentiate into root hair cells and non-root hair cells under the guidance of spatial cues. Previous studies have characterized a transcription factor network that ensures the fidelity of this spatial signal interpretation (Bruex *et al.*, 2012). At the same time, a growing number of phytohormones have been found to be required for normal root hair patterning and morphogenesis (Pitts *et al.*, 1998; An *et al.*, 2012). However, it is still largely unclear how these phytohormones crosstalk with fate determination factors and how the different phytohormones might interact. In this study we sought to bridge these gaps between cell fate determination, phytohormone functioning, and their interactions.

### The role of phytohormones in regulating root hair initiation

Two aspects of spatial regulation are involved in Arabidopsis root hair initiation (Bruex *et al.*, 2012). First, root hairs mainly form at the maturation zone, where root epidermal cells acquire their final cell features. This phenotype suggests the onset of root hair initiation is tightly associated with root development. Second, root hair cells are always located above two cortical cells, which are controlled by positional cues (Bruex *et al.*, 2012; Grierson *et al.*, 2014). Among the phytohormones that we studied, only ethylene plays a dual function in root hair initiation and elongation, whereas auxin and cytokinin mainly affect elongation.

When treating the seedlings with exogenous auxin, ethylene, and cytokinin, we found that only ethylene treatment enhanced root hair initiation in the wild-type line and the hairless mutant *cpc* (Fig. 1B, C). Root hair initiation was inhibited when ethylene biogenesis was blocked (Fig. 1B). However, epistatic analysis has indicated that auxin and ethylene function after the fate determination of root epidermal cells (Masucci and Schiefelbein, 1996). Although it is still unclear how exogenous ethylene promotes the formation of ectopic root hairs, we showed that ethylene treatment activated the expression of genes that function in root hair initiation even in the *cpc try* mutant, a line with very limited root hairs (Fig. 2E).

Interestingly, treatment with exogenous ethylene mainly induced root hair initiation at the H positions in the hairless line *cpc*, whereas the ethylene biogenesis inhibitor significantly reduced root hair density equally at the H and N positions in the hairy lines *ttg1* and *gl3 egl3* (Fig. 1C, D). This result suggests that H position cells have a higher tendency to form root hairs even when the *CPC* gene is mutated. One tempting explanation is that the homologues of *CPC*, such as *TRY* and *ETC1*, are still functional (Schellmann *et al.*, 2002; Kirik *et al.*, 2004). In contrast, when WER (a position-dependent regulator of epidermal cell patterning) is dissociated with GL3, EGL3, and TTG1, inhibition of root hair initiation is released in both positions, which makes the N and H position cells behave similarly in the *ttg1* and *gl3 egl3* mutants when ethylene biogenesis is blocked (Fig. 1D) (Lee and Schiefelbein, 1999).

Additionally, we observed that mutants for epidermis fate determination regulators were less sensitive to phytohormones with regard to root hair elongation (Fig. 2C). The expression level of many root hair-specific genes was also altered in those mutants (Fig. 2D). Interestingly, the *ttg1* mutant shows enhanced root hair initiation but normal hair length (Figs 1D and 2C). Given that similar genes are involved in both initiation and elongation, their actions may also be subject to another layer of spatial regulation (Jones *et al.*, 2002; Yi *et al.*, 2010). Collectively, these observations support the hypothesis that cell reprogramming provides a scenario for the normal function of phytohormones.

### Partially redundant functions of different phytohormones in root hair elongation

Antagonistic activities of phytohormones are one of the key mechanisms to ensure the robustness of regulation, and have been found in root and shoot meristems (Wolters and Jurgens, 2009). Surprisingly, all the phytohormones that we tested were able to promote root hair elongation (Fig. 2A, B). Strigolactone, another recently reported hormone, also promotes root hair elongation (Kapulnik *et al.*, 2011). From an evolutionary prospective, this coincidence might suggest that elongated root hairs tend to benefit plants under different environments. On the other hand, more analysis may be needed to reveal which hormones play the more predominant role under different physiological conditions.

Notably, all of the phytohormones examined can regulate root hair elongation independently. For example, Pitts *et al.* (1998) showed that exogenous auxin induced root hair elongation in the absence of an ethylene signal. We also observed that the cytokinin could promote root hair elongation independent of auxin and ethylene (Fig. 3A, B). Auxin and ethylene also enhanced root hairs in the cytokinin enzyme overexpression line (Fig. 3C). However, phytohormones also regulate the biogenesis of each other, which adds another layer of regulation to the network. It has been documented that cytokinin can enhance the production of ethylene in roots (Chae *et al.*, 2003; Hansen *et al.*, 2009). We also observed that exogenous cytokinin induced root hair genes slightly differently in the wild-type and *ein3-1* lines (Fig. 4C–E). Future studies on hormonal interactions under different physiological conditions might provide substantial new insights into the network.

### Auxin, ethylene, and cytokinin target similar genes in regulating root hair elongation

Although auxin, ethylene, and cytokinin do not rely on each other in root hair elongation, our data indicate that these hormones induce the expression of a similar set of genes to promote elongation (Fig. 4A–C). Microarray data analysis revealed that a substantial portion of root hair-specific genes were co-regulated by two or three hormones (Fig. 4A, B). Using stringent criteria of genes with the GO:0048765 annotation and that have the RHE in their promoter, we identified nine ‘core genes’ mainly expressed in root hair cells and positively regulated by auxin, ethylene, and cytokinin (Fig. 4A–C). Among the protein products of these genes, EXP7, EXP18, PRP3, and RHS10 are known to act at the cell wall, indicating that cell wall remodeling is one of the key processes that is regulated by these phytohormones (Bernhardt and Tierney, 2000; Cho and Cosgrove, 2002). Additionally, the loss of function or over-expression of most of these genes causes defects in root hair morphogenesis (Bernhardt and Tierney, 2000).

Considering that different hormone signaling ultimately regulates a group of transcription factors, the identification of key signal mediators would help us to understand the crosstalk networks. Gene expression profiling data suggest that RSL4 might be one of these factors. First, RSL4 expression could be stimulated by exogenous auxin, ethylene, and cytokinin (Fig. 4D) and, in contrast, its expression is repressed in the ethylene signaling mutant *ein3-1* (Fig. 4E). Second, nearly all of the hormone-regulated ‘core genes’ are positively regulated by RSL4 (Yi *et al.*, 2010). More importantly, a recent study has suggested that pulse expression of RSL4 controls root hair size (Datta *et al.*, 2015). Other mediators may also exist. For example, another transcription factor ZFP5 also integrates ethylene and cytokinin signals to promote root hair elongation (An *et al.*, 2012). We believe that further characterization of these signal mediators and their target genes will help us to understand how phytohormones facilitate root hair elongation.

## Supplementary data

Supplementary data are available at *JXB* online.

Table S1. Primers used in this study.

Figure S1. The *aux1* mutant is insensitive to exogenous auxin and ethylene.

Figure S2. Exogenous auxin and cytokinin mildly induce root hair elongation in hairless mutants.

Figure S3. The short-hair phenotype of 35S:CKX2 mutant can be reversed by cytokinin oxidase-insensitive cytokinin BA.

Supplementary Data
