# Supplementary material for: Multiple phytohormones promote root hair elongation by regulating a similar set of genes in the root epidermis in Arabidopsis
Source: J Exp Bot. 2016 Oct 31;67(22):6363–72. doi: 10.1093/jxb/erw400 (PMC5181580; doi:10.1093/jxb/erw400)
Supplement: Supplementary Data [file supp_67_22_6363__index.html]

Multiple phytohormones promote root hair elongation by regulating a similar set of genes in the root epidermis in Arabidopsis — Multiple phytohormones promote root hair elongation by regulating a similar set of genes in the root epidermis in Arabidopsis — Supplementary Data 

# Multiple phytohormones promote root hair elongation by regulating a similar set of genes in the root epidermis in Arabidopsis

## Supplementary Data

Data files

- supplementary\_table\_S1\_figure\_S1\_S3.pdf - Supplementary Data
