# Supplementary material for: Multiple phytohormones promote root hair elongation by regulating a similar set of genes in the root epidermis in Arabidopsis
Source: J Exp Bot. 2016 Oct 31;67(22):6363–72. doi: 10.1093/jxb/erw400 (PMC5181580; doi:10.1093/jxb/erw400)
Supplement: Supplementary Data [file supp_erw400_supplementary_table_S1_figure_S1_S3.pdf]

Supplementary Table S1. Primers used in this study

Gene name	Accession	Direction	Sequence
ACTIN2	AT3G18780	Forward	AACCCAAAGGCCAACAGAGA
		Reverse	AAGGTCACGTCCAGCAAGGT
EXP7	AT1G12560	Forward	AACCATGGGTGGTGCATG
		Reverse	CCGCATCCGTAACCATCA
EXP18	AT1G62980	Forward	CAGCGCAACTATGGGTGG
		Reverse	ATGGACTCGTGGCGGATT
RSL4	AT1G27740	Forward	ACAAGACAAGAGCTTGCG
		Reverse	ATCAGTGGCTGTCCCTTT
RSL2	AT4G33880	Forward	CGTGCCACATCTACTGATAAAAACA
		Reverse	TTCTCCTCCACCGTCTTG
RHD2	AT5G51060	Forward	GAATGCATAGGAATGACTT
		Reverse	CAGAAGCACTAAGACTGATAA
RHD3	AT3G13870	Forward	TCCACAAAGTAGTGGGAAGA
		Reverse	GTTTCTCGAAAGCAGTATCA
RHD6	AT1G66470	Forward	CAAACGTTCTCACACGGGAGAG
		Reverse	GGTGCCATTGGGAACAAG
RHL1	AT1G48380	Forward	ATTTCCCACAGGGTCGTA
		Reverse	CTTTTGTCCCAATCCACC
RHL2	AT5G02820	Forward	AGCTTTTCCAGGACCAGA
		Reverse	CCCATTCCCATCTTTGTG
XTH13	AT5G57540	Forward	GTCACTGCTTACTACCTTTCATC
		Reverse	TTTCACGGTTACCTTTGC
ROP2	AT1G20090	Forward	CTTTTCCTACTGATTATGTGC
		Reverse	AAGATCGAGTTTTGTCCC
PRP3	AT3G62680	Forward	ACCCTATTCTAGGAGCAAA
		Reverse	CAACGACAAGTGGAAGTATC
MRH6	AT2G03720	Forward	AACACCCGTAGGACAAGC
		Reverse	TCTGCCCTAACACCAAAA
RSH4	AT1G30850	Forward	AACGATAATGAGTGGCAAGG
		Reverse	AGCATAGGAAGCGGAACG
RHS10	AT1G70460	Forward	CTTCTCCATCAAATCAGATGGATTC
		Reverse	CTTCTGTTATGTCCGTTAGC
RHS16	AT4G29180	Forward	AGGCCAAGCAGGTTTCAT
		Reverse	CGGGTAACCGTATTCTTCA
RHS18	AT5G22410	Forward	TTGTTTTAGCAACGGGTGT
		Reverse	TCAGCACAAGAAACGACC
AT3G12540	AT3G12540	Forward	TTCTTCCTCCTCAGGTTCA
		Reverse	CCATAACTCTGGGACTTTGA
CSLD3	AT3G03050	Forward	TTAGTCCTTACAGGCTTTTG

Continued

		Reverse	TGCTCGGTGTAGGTGTTT

PRP1	AT1G54970	Forward	CTATTCAAGGAGCGAAAGC
		Reverse	GTTGACATTGGTTGGGTTC
LRX1	AT1G12040	Forward	AAGCAGACCACGACGATG
		Reverse	ATGAAGGAGAAGGAGCACA
COW1	AT4G34580	Forward	CCACATGATGCTTCGATTTTTGAG
		Reverse	TAGCCTTGAGGGTAGTGC
TIP1	AT5G20350	Forward	CAGTACCTTATCGAGCATGG
		Reverse	CGTGACAAAGAAAAGCAGT

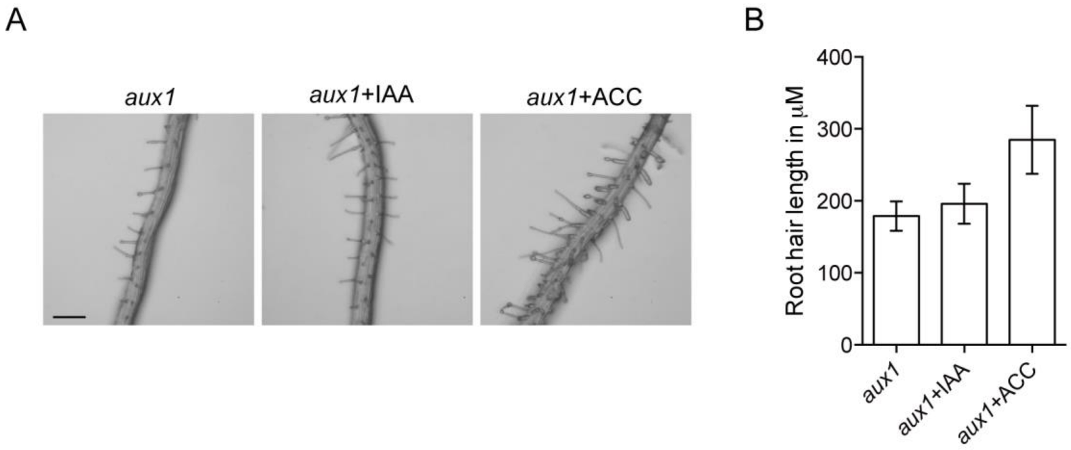

Supplementary Figure S1. *aux1* mutant is insensitive to exogenous auxin ethylene. (A) Images of root hair region of *aux1* seedlings grown on MS plate or MS plate supplemented with IAA or ACC are shown. Scale bar=200μM. (B) Average root hair lengths of *aux1* seedlings grown on MS plate or MS plate supplemented with IAA or ACC are shown. Error bar: ±SD.

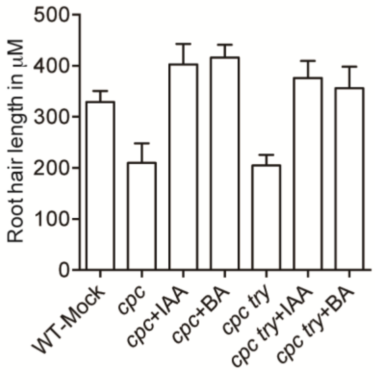

Supplementary Figure S2. Exogenous auxin and cytokinin mildly induce root hair elongation in hairless mutants. The average root hair lengths of different lines under treatments were determined. Error bar: ±SD.

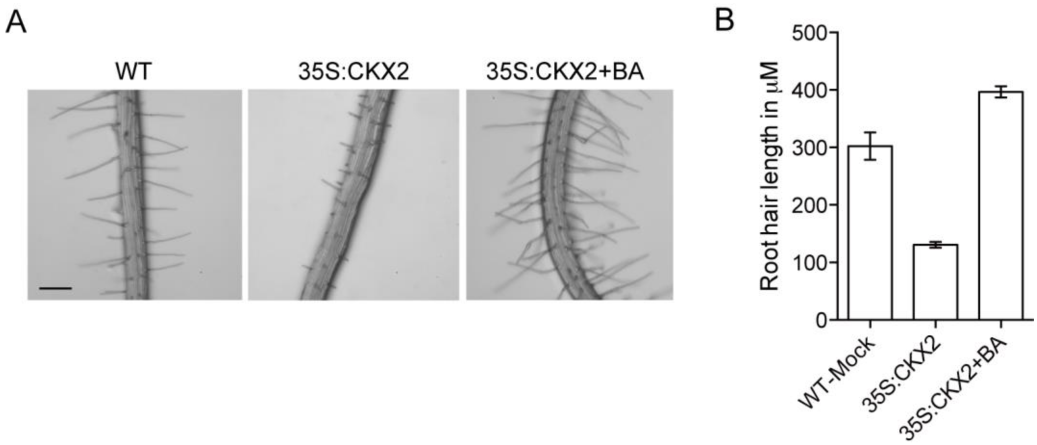

Supplementary Figure S3. The short root hair phenotype of 35S:CKX2 mutant could be reversed by cytokinin oxidase-insensitive cytokinin BA. (A) Root hair phenotypes of wild-type, cytokinin oxidase overexpression line 35S:CKX2 and 35S:CKX2 line treated with exogenous BA are shown. Scale bar=200μM. (B) Average root hair lengths of seedlings shown in (A) are presented. Error bar: ±SD.
